# Constructing democratic participation in welfare transitions: An analysis of narrative interactions

**DOI:** 10.1111/hex.12970

**Published:** 2019-11-06

**Authors:** Mare Knibbe, Klasien Horstman

**Affiliations:** ^1^ Department of Health, Ethics and Society Inequality Participation Globalisation (IPG) Faculty Health, Medicine, Life Sciences Maastricht University, Care and Public Health Research Institute (Caphri) Maastricht The Netherlands

**Keywords:** care theory, democracy, narrative analysis, participation society, public discourse, welfare reforms

## Abstract

**Objective:**

This article provides insights into the democratic character of local enactments of welfare reforms by analysing narrative interactions about changes in care. We analyse processes of storytelling that are part of the interactions between citizens giving, receiving and organizing care and the policymakers governing welfare reforms. We also study how narrative interactions shape understandings about changing care practices and what types of narrative interactions support democracy in care.

**Background:**

Stories about recent welfare reforms include messages about citizens’ care, citizen participation, citizens’ powers and revitalization of democracy. However, researchers have cast doubt on their emancipatory and democratic character.

**Research setting and methodology:**

We conducted research of four initiatives and municipal policy settings in the city of Maastricht that organized social care in lifeworlds connected to arts, crafts, farming and entrepreneurship during welfare reforms. Using narrative ethnography, we analyse narrative interactions between the master narrative of welfare reforms about ‘lifeworld care’ and ‘citizen powers’, and small stories told by participants in new care practices.

**Results:**

We identified two types of narrative interaction: idealizing and pragmatizing. Idealizing narrative interactions were strategic for care initiatives in finding support and for policymakers in proving that a so‐called ‘participation society’ works. Pragmatizing narrative interactions gave expression to insights into the everyday practices of social care experiments and included a greater variety of stories.

**Conclusions:**

We conclude that pragmatizing narrative interactions adjust the master narrative about welfare reforms and replace ideals of independency with ideals of active participation in webs of dependency and care.

## 1 INTRODUCTION

Political stories about transitions from welfare state to ‘participation society’ in the Netherlands or a ‘big society’ in the UK have promoted the reallocation of care work and various vulnerable groups from bureaucratic professional organizations to the everyday lifeworlds and volunteers. These transition narratives include an emancipatory storyline about liberation from bureaucratic dominance that will revitalize democracy through new forms of citizen participation.^1^, ^2^ Nonetheless, the democratic legitimacy of these welfare reforms has been questioned.^3^, ^4^, ^5^ In the UK, for example, the influential transition narrative of big society claimed to be about empowerment, emancipation of vulnerable groups, and civil society. Bulley and Sokhi‐Bulley argue, though, that it was in fact instrumental to management and control.^3^ It was about ‘the power to cope with neoliberalism rather than challenge it’ (p. 465).^3^ The decline of civil engagement during the big society programme also suggests that the revitalization of democracy has failed.^4^ In the Netherlands, meanwhile, scholars like Horstman and Putters have pointed out that welfare reforms were carried out in a context of declining turnouts during elections, especially by the groups most affected by these reforms.^5^, ^6^ They argue that more emphasis is needed on participation in political decision making and less on participation forms that are instrumental to predefined government goals. Bovens, Wille and Van Reybrouck diagnose the democratic deficit in terms of ‘diploma democracy’.^7^, ^8^ Groups with lower levels of education have difficulties presenting themselves in the public sphere and are not part of the elected representation in government, which is dominated by politicians with an academic education. These studies lead us to question the emancipatory and democratic character of welfare reforms: political transition narratives do not represent the experiences of people most affected by the reforms.

On the other side, critics of welfare regimes point to the democratic potentials of reform.^9^, ^10^ Even if research about big society shows a decline in democratic participation, reforms may promote participatory democracy in other settings. Jim Diers, for example, promotes ‘asset‐based community development’, an approach based on critiques about ‘welfare colonialism’, and argues that self‐organizing communities in Seattle have revitalized participatory democracy.^11^ In the Dutch context, researchers have predicted a strengthening of participatory democracy when welfare institutions relinquish some of their power.^12^ These researchers see the old bureaucratic welfare institutions as a hindrance to citizen‐initiated change. During Dutch welfare reforms, research institutes and the government discussed citizen engagement in new forms of care in terms of ‘do‐democracy’, which can be seen as a form of participatory democracy.^13^ The government saw this as a route for residents to have a say in the shaping of society by engaging with concrete issues in the public domain.^14^ To stimulate the do‐democracy, municipal and national governments and research institutes issued many publications about successful citizen initiatives with stories demonstrating the power of citizen collectives.^10^, ^15^ However, scholars analysing the new forms of public participation or do‐democracy have pointed out that this may increase inequalities and thereby undermine democracy.^16^, ^17^ Calhoun, for example, points out that the growing participation ideology is a mixed blessing: it has managed to involve citizens in large‐scale political processes in new ways, but it also disguises the continued reproduction of inequalities.^18^ In the Netherlands, a number of scholars who have analysed do‐democracy are not convinced about its democratic character.^2^, ^19^, ^20^ According to them, citizen actions are often initiated top‐down and do not arise spontaneously in a bottom‐up process. Moreover, most influential citizen initiatives consist of high‐income citizens and not of vulnerable low‐income groups.

Other experiments with democratic renewal during welfare reforms worked with deliberative forms of democracy. In ‘citizen summits’ or ‘mini publics’, citizens were selected based on random or purposeful selection and invited to deliberate about public issues.^5^, ^8^, ^21^ In well‐organized conversational settings supporting respect and listening, citizens deliberate and may form and alter their opinions without the pressures of the media and electorate faced by elected representatives. The outcomes of such deliberations have been embedded in various ways in institutionalized political processes as recommendations or starting points and support for new citizen initiatives. While analyses of such deliberative democracy show that citizens engage in respectful dialog and bridge social and cultural divides, such deliberative approaches also have an exclusionary character. As Polletta points out, ‘deliberators’ are expected to support their arguments with reasons that are acceptable to others.^22^ People in marginalized positions often do not have the discursive resources to convince others, however. ‘The danger, then, is that already marginalized speakers and positions are further marginalized’ (p. 224).^22^

These debates about democracy in the contexts of care and inequality show the gains and limits of citizen participation in do‐democracy and deliberative forms of democracy. In spite of their potential, different forms of democratic renewal in the context of welfare reforms are at risk of reproducing or aggravating social inequalities. Moreover, the debates highlight that social and political inequalities cannot be separated: in stratified societies, there are no ideal communicative settings in which citizens can deliberate as equals regardless of existing social inequalities.^23^ In this article, we aim to contribute to this debate by analysing the discourse that emerges within narrative interactions in the new care practices that developed during welfare reforms. This means that we follow the processes of storytelling between citizens giving, receiving and organizing care and the policymakers governing welfare reforms. When compared to deliberations as a specific type of argumentative discourse, narratives form another type of discourse in which people organize and connect experiences and events.^24^ In the context of debates about democracy and welfare reforms, narrative interactions are interesting because they include fragmented, incomplete, personal and small stories as well as more articulate and coherent storylines about the past and future of care practices. Moreover, in narrative interactions, narrators make sense of their own experiences and observations by connecting to other stories. Narrative interactions therefore have the potential to include marginal perspectives and experiences in the formation of public understandings about welfare reforms.

We analyse narrative interactions about new practices of care with a focus on two questions: (a) How do narrative interactions shape understandings about changing care practices? (b) What types of narrativ1e interactions support democracy in care? We first discuss scholarship about narrative interaction in relation to democracy in care. Next, we introduce our case, welfare reforms in a Dutch city. Subsequently, we present our findings about narrative interactions in new practices of care.

## 2 STUDYING DEMOCRACY IN CARE THROUGH NARRATIVE INTERACTIONS

We build on the work of Gubrium and Holstein in narrative ethnography to analyse narrative interactions and their meaning for democracy in care practices.^25^ Advocates for democracy and emancipation look with suspicion at stories told* about *vulnerable groups or individuals and prefer to include stories told *by* people in subordinated, exploited or otherwise disadvantaged positions.^23^, ^26^ Nonetheless, it is common practice within care and policy‐making to recycle and retell stories *about* groups such as ‘the unemployed’, ‘the homeless’, or ‘impaired people’.^27^ Moreover, in care practices the telling and retelling of stories about needs, vulnerabilities, recovery and resilience are a vital part of caring interactions.^28^ Even if the ideal is that people in care tell their own stories, we also need a better understanding of the democratic meaning of such narrative interactions where people retell stories of and about others in different settings.

We use the concept of master narratives as explained by Lindemann Nelson. According to her, transition narratives about welfare reforms can be seen as ‘master narratives’, stories ‘that serve as summaries of socially shared understandings’ (p. 6).^29^ Master narratives play a role in shaping identities and relationships, and their plotlines guide images of change. Master narratives are often resisted in counter‐stories oriented at changing specific relationships or identities. Lindemann Nelson gives the example of hospital narratives about ‘technical’ doctors and ‘touchy‐feely’ nurses.^29^ This presentation of the division of labour is nourished by master narratives about rational men and emotionally subservient women. From many small stories, anecdotes and histories, nurses constructed a counter‐story that moved beyond this rational‐emotional dichotomy and showed that nurses were skilled professionals. However, often small stories and adjustments to master narratives are often not explicitly told as counter‐stories and remain unheard by broader publics.^16^ A way of tracing how the voices of relevant actors are heard, obscured or translated during welfare reforms is to analyse narrative interactions between master narratives and everyday small stories about new care practices.

In narrative research, several authors have argued that narratives do not develop in a vacuum but are part of everyday narrative interactions in diverse settings and relationships.^24^, ^25^, ^30^ Gubrium and Holstein call this the ‘narrative environment’: the intimate and distant relations, organizations and social settings in which stories are told or remain untold and are connected to other stories.^25^ Intertextual connections between stories told in different narrative environments can be traced through ethnographic study. While much narrative research has focused on the internal structuring of narratives, recent contributions have paid more attention to what narratives *do*, to their performance and ‘external organization’.^25^, ^31^

Narrative interaction thus refers to intertextual connections between small stories, master narratives and the performance of narratives in shaping understandings about care, thereby preparing decisions during welfare reforms. For debates about democracy, it is interesting to examine these narrative interactions because they include many people in marginal positions who do not sit at roundtable discussions where decisions are made about allocations of resources for care. Moreover, it is within these narrative interactions that understandings are formed about *who* is giving and receiving care. Small stories told in informal settings by people in vulnerable positions can be retold, translated or ignored in more formal policy‐making settings. Thus, such narrative interactions can support or hinder inclusion in policy‐making.

### 2.1 Research setting and data collection

We studied a specific Dutch case, namely welfare reforms in the city of Maastricht, a city of about 120 000 inhabitants in the Netherlands. Welfare transitions in Maastricht were particularly challenging as this post‐industrial city faces a high burden of disease and unemployment compared to other regions.^32^ We analysed transition narratives in the interactions between municipal policymakers and social care initiatives, selecting four initiatives that the municipality had identified as good examples of social care befitting the participation society.

The four initiatives had different historical, organizational and social backgrounds. They included two citizen initiatives ‐ a community arts centre (A) and a children's farm (B) ‐ an entrepreneurial citizen initiative that engages entrepreneurs and companies in the development and care for people with disabilities (C), and one initiative with roots in a publicly funded professional care organization for people with intellectual disabilities, now transforming into a more entrepreneurial organization (D). These initiatives grew in volume and changed in character after January 2016. Two legal reforms account for these changes in part. First, the Societal Support Act (Wmo) describes the care obligations of citizens, households, informal networks and public facilities.^33^ Under this law, state‐funded support is considered a last resort as family, friends and social initiatives are expected to provide mutual help, thereby preventing the need for individual state‐funded support. Second, the Participation Act prescribes forms of reciprocity for welfare benefits, reintegration into the labour market, and obligations to facilitate the participation of people with disabilities in the labour market.^34^

To analyse the narrative interactions, we conducted ethnographic research between January 2016 and July 2017, with occasional follow‐up in 2018. The ethnographic research consisted of participant observations and conversations recorded in detailed field notes, audiotaped semi‐structured interviews and analysis of policy documents (see Table 1).^35^ The first author, supported by two students, conducted participant observation for 20 months in three categories of ‘narrative environments’: first, the daily work of participants in workplaces, farming, art workshops or other social meetings; second, in regular meetings of the leadership of each social care initiative; and third, in municipal policy meetings, including meetings between leaders of social care initiatives and municipal policymakers (see Table 2). To collect stories from municipal policy environments, we selected landmark documents dating from the years 2015 to 2017, during which the Maastricht welfare policy was developed and explained. We also attended political meetings and conversations with municipal policymakers that funded the four social initiatives. These three types of narrative environment were systematically included. However, stories told by the professionals of referring institutions were also included occasionally. In each of the four initiatives, we interviewed at least ten and at most 21 people for whom participation in the social care initiative was part of a personal struggle with health, employment or social reintegration. We held interviews with 12 leaders of the social care initiatives, three municipal policymakers who were responsible for supervising the funded initiatives, and four consultants at the social security office who were responsible for enacting the Societal Support Act and the Participation Act and who referred people to the social initiatives. Respondents were asked to talk in their own words about the meaning of the social care initiative in their lives and in the field of care and support. The interview approach further enabled the study of ‘intertextuality’ as respondents were asked to comment on stories picked up in other conversational settings.

**Table 1:** Data collection

	Initiative A Community arts centre	Initiative B Children's farm	Initiative C Social entrepreneurial citizen initiative	Initiative D Care organization that became an entrepreneurial initiative	Partners of initiatives in the municipality and other organizations	Total
Interviews & conversations by first author	10	13	18	17	7	
Interviews by student^a^	‐	6	3	6	‐	
Total interviews	10	19	21	23	7	80
Extended periods of participant observation (Intensive participant observation by student)	December 2016‐July 2017	June 2016‐July 2017 (April & May 2017)	January 2016‐May 2017	Feb 2016‐July 2017 (May‐July 2017)	January 2016‐July 2017	20 mo

a Two students, working on their bachelors thesis for social work and for health sciences contributed to the data collection of this project. They had both followed training in qualitative research‐methods and their fieldwork was supervised by the first author. The students formulated their own research aims and question, however their questions also contributed to the overarching research project.

**Table 2:** Narrative environments and central topics

	Narrative environments	Central topics in interviews & observations
First author	**Daily work interactions: **Creative workplaces (A), woodwork place (D), Canteens (A, B & D), animal care (B), farm café (B), volunteer work meetings (A & B), community gatherings (C), services & care points (C), social meeting places of partner companies of C **Leadership meetings: **Regular meetings of board/leaders in A, B C, &D **Policy documents and meetings with municipal officials and professionals from other organizations: **Leaders of A, B, C, D Municipal officials working on welfare reforms Consultants for Societal Support Act & Participation Support Act Professionals of mental health organizations Professionals of unemployment agency Entrepreneurs & employees of partner companies	Personal trajectories with work, (un)employment, health, & disability that brought participants to the initiative Social contacts, care and work in initiative Health and well‐being in initiative Relations between initiative and other care organizations
Student social work	**Daily work interactions: **Services & care point C Café of D Canteen and other meeting places of D	Initiative C Experiences with multiple care organizations in the life of the participant Perception of caring needs Perception of care received The meaning of initiative C Initiative D Meanings of participation in initiative Self‐management & powers of participants with disabilities Role of professionals
Student health sciences	**Daily work interactions: **Green maintenance work (B) Animal care (B) Farm shop (B) Farm café (B)	Personal experiences with initiative B Influences of B on health: Eating Physical exercise Other health‐related experiences

We chose a participatory research approach to support collective learning processes about the transition of welfare and care arrangements.^36^ Design of fieldwork and results was discussed with leaders of the initiatives and the municipality. Participatory ethnographic design was guided by the code of conduct in anthropology.^37^ We were clear about our research aim in all interactions in the field, using information letters and oral explanations.

The focus of analysis was on the external organization of stories: on the types of formal or informal encounters in which stories were told and the intertextual connections between small stories and master narratives told by participants and leaders of the social care initiatives. As a demonstration of this approach, here is the story of a participant of initiative C, Mr Pamuk, which was told in different narrative environments. Mr Pamuk said in an informal setting that he is angry about being in the Netherlands because he would much rather live in Turkey, his country of origin. In a meeting that the leaders of C have with the unemployment agency, they retell this story while at the same time connecting it to other stories about medicalization, labelling and the power of genuine personal contact. One leader says:

We believe that something big happens in a real conversation in which you can talk about what you really want, and we are very critical about labeling. For example, we talked to a young Turkish man who received the label of autism. But we believe that he is angry because he does not live in Turkey.

In this narrative interaction, the leader of C makes intertextual connections and expects policymakers at the unemployment agency to make their own connections and consider stories about autism in different ways. Moreover, by retelling this small story in the narrative environment of an unemployment agency, the leader of C also gives the small story the power to challenge the bureaucratic system of the agency, which includes medical labels. These narrative interactions can include complete stories, but they also include small and fragmented utterings, like Mr Pamuks expression of anger, that acquired additional meaning in interconnected narratives. In our analysis, we trace these types of narrative interactions.

## 3 RESULTS

**Figure 1:**
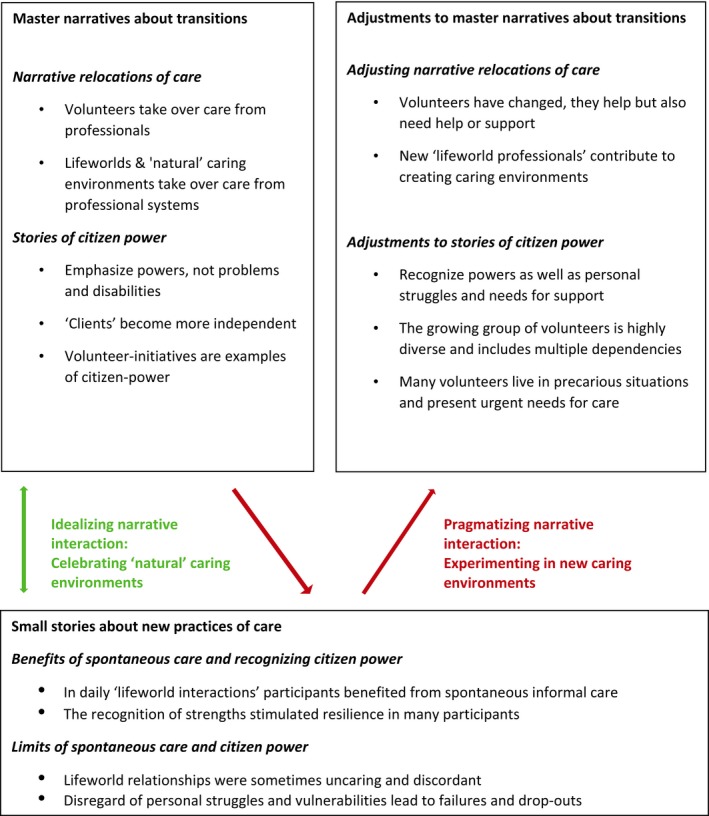
Idealizing and pragmatizing narrative interactions

We identified two types of narrative interaction between master narratives about the transitions and small stories told in newly developing care practices. The first type can be characterized as ‘idealizing narrative interactions’, in which small stories about care were selectively retold to support the policy ideal of caregiving within informal lifeworld relationships. The second type of narrative interaction gave more prominence to small stories that did not fit the master narratives. We refer to them as ‘pragmatizing narrative interactions’. Here, policymakers and citizens used small stories told in care practices to make adjustments to the caring roles and relationships imagined in the transition narratives. Figure 1 gives an overview of the two types of narrative interaction and adjustments to master narratives. Below, we first outline idealizing narrative interactions, and then we describe pragmatizing narrative interactions. In the discussion, we reflect on the democratic character of these narrative interactions.

### 3.1 Idealizing narrative interactions

In idealizing narrative interactions, the master narratives about participation society remained largely unchanged. In official policy settings, leaders and municipal policymakers retold a selection of the small stories provided by new social care initiatives that added *couleur locale* and lively examples. The small ‘counter‐stories’ that did not fit in this master plot were not integrated in idealizing narrative interactions and remained local, incidental and isolated. Consequently, small stories that could potentially change shared understandings about welfare reforms remained powerless.

#### 3.1.1 Narrative relocations of care

A first storyline in master narratives is dedicated to the relocation of care from professional institutions to citizens and volunteer initiatives. The vision of the municipal future agenda was to move caring activities closer to lifeworlds: ‘We put the citizen and their direct environment at the centre. And not the system’.^38^ The contrast between lifeworlds and systems was supported with metaphors borrowed from nature:

We stimulate voluntary engagement and value volunteers. Volunteer initiatives have a natural way of making people look after each other. They are the unique DNA of our municipality, center, or neighborhood because they originated from the energy of our residents and not from blueprints.^38^

In the context of this policy narrative, the social care initiatives were funded as they were considered to be locations of ‘collective power’, with volunteers and ‘lifeworld care’ that could help prevent the need for more specialized and expensive care. The municipal budget plan announced for 2016:

The fund will finance citizen initiatives that have the potential to take over a social task of the municipality and to subsequently execute this task cheaper and in a qualitatively better way.^39^

Participants in the initiatives told many small stories about mutual care between volunteers that support these master narratives. In initiative A, a woman who was heavily traumatized in her youth made a wedding dress promising love for herself. She said that she found friends and emotional support when taking part in the initiative:

I feel that I radiate, I am happier, have contact with people, and I can develop. And I am grateful because I have to give something back… When they need me, I am there for them.

Volunteers also recounted how they helped other vulnerable volunteers join in daily work and how they were attentive to their sensitivities. A volunteer at the farm talked about a man that she took along to care for the animals:

…he can spontaneously become completely downhearted. And yes, I feel able to be there for that man, yes. I mean, I recognize a part of myself because I also have that problem. And yes, I can deal with it, and he likes it.

While these small stories resonate with the master narratives about ‘natural care’ or ‘lifeworld care’, participants and staff members of the initiatives or partner companies were sometimes overwhelmed or surprised by the caring needs of participants. A driver of the transport company that collaborated with initiative C told in a conversation in his canteen about a participant of C who joined him on his transport route:

Yes, he [participant of C] needs a lot of attention… In recent years I have taken more participants from the initiative on my route… One time it was a person who needed a lot of attention. With my last delivery I had asked him to stay in the car because I wanted to finish quickly. When I came back, he had disappeared, and I couldn’t find him. I called the leader of C. A week later we heard that the police had found him.

The driver made clear that he was enrolled in caring activities because his boss supported the social entrepreneurial ideal and that he experienced providing care as very difficult. This small story had the potential to challenge the master narrative about mutual care in the everyday lifeworld as it shows that the expectation of mutual care cannot be fulfilled. However, in this case the small story did not challenge nor change the master narrative because it was not retold in other narrative environments. In idealizing narrative interactions, only the small stories that demonstrate successful forms of lifeworld care are retold in the narrative environments of leaders and policymakers. This small story about the difficulty of providing adequate care in the lifeworld of regular transport work remained an isolated and local exception that was not given the power to change shared understandings about welfare reform. Thus, idealizing narrative interactions hindered the construction of democracy in this new care practice.

#### 3.1.2 Stories of citizen power

A second storyline in master narratives about the welfare transitions expressed the importance of recognizing ‘citizen power’. Like national and international examples, municipal policymakers supported the celebration of citizen powers:

We expanded the concept list with the terms ‘self‐sufficiency’, ‘own strength’, ‘informal care’, and ‘citizen power’. The term ‘client’ will only be used in the future agenda for the notion of ‘client support’.^27^

Consultants for the Societal Support Act (Wmo) and Participation Act were instructed to stimulate ‘citizen power’ instead of assigning professional support, and their stories show how they identified citizen power at the new social care initiatives:

We look at what is necessary… to move someone to empower them and to make them experience wellbeing… someone does not need continuous supervision. If I look at this neighborhood, there are a lot of facilities like the community art center (initiative A) and a lot of volunteers… where they can have a place, drink coffee, play a game, or talk… (Wmo consultant)

This master narrative resonated in many small stories told by participants on the work floor. Miss Lowenthal's personal story, for example, was retold in various social meetings and policy meetings of initiative C as an example of how someone left her client role behind and developed new strengths. Lowenthal received an allowance because she was considered disabled on the basis of ADHD and other psychological problems. However, when she became active in initiative C, she quickly developed into a location manager for a ‘services and care point’. A services and care point is a new kind of shop that creates entrepreneurial opportunities for citizens with long‐term unemployment or disability (by providing services such as computer support or tailoring) in combination with a social meeting point where citizens in the neighbourhood can ask questions about care and support. Lowenthal explained how initiative C had changed her life:

I receive an allowance (Wajong), and I was waiting to hear about reintegration, but I heard nothing, so I decided to call them myself… because, yes, sitting at home… it made me really depressed… The stability that the allowance gave me at first really helped me, but no this is not what I want with my life… The unemployment agency (UWV) had just started a pilot with initiative C, so I contacted them… and the best thing about initiative C is that they start by removing the labels you received… it’s not like ‘so you have ADHD or are Borderline’. They do not think everyone is the same, but they look at what kind of person you are and not at your ‘baggage’ (diagnosis)… And indeed, I found out that sometimes I can push my boundaries, and that feels good.

Her story was retold at several public meetings of initiative C as a good example. Moreover, Miss Lowenthal joined in an official meeting at the municipality about municipal collaborations with the services and care point. In this meeting, a municipal official said to her, ‘the power of this project is that you are its public face’.

The municipality decided to support the services and care point, by embedding it in various other infrastructures for care and support, such as a new ‘social care team’. This shows how personal stories like that of Miss Lowenthal became meaningful in various social and policy settings as demonstrations of citizen power and ‘personal strength’.

By contrast, many personal stories that showed powerlessness or failure were not retold in various narrative environments. One example is the story of Mr Perry. Mr Perry talked about his relentless attempts at finding paid work as he feared his disability allowance might stop soon because of welfare reforms:

I worked for 13 years in a security company (mediated by a social enterprise), but during the financial crisis they fired me. And since then I’ve been at home… it has been seven years and I have done over 2,000 job interviews… for me, paid work has highest priority, because with the government plans I will go from 900 euro per month to 500 per month (disability allowance) and that is not much… Initiative C has workplaces without payment, where you can keep your allowance. They place people somewhere and then they hope that the employer will decide after some time to offer the person paid employment

• But in your case, this didn't happen
‐ No
• If you look at your strengths and weaknesses, how do you look at that? Did you have a conversation with initiative C about this?
‐ Yes, they know that I’m able to help myself… and that I can articulate my own case and that kind of thing, that I keep myself busy.

With this story about his strengths and relentless job search, it was difficult to understand why Perry did not manage to find employment. The leader of C explained in a private conversation that she did not manage to convince Perry that an unpaid position might also be an acceptable achievement for him. It seems that no one managed to put his ‘strengths’ in perspective, to reflect on the failing strategy of the ongoing job search or take away his financial worries. Like the story of the driver, this story also had the potential of a counter‐story, challenging shared understandings about citizen power in welfare reforms. However, this small counter‐story about failure also had a similar lifecycle and remained an isolated case. It was not retold in meetings between leaders of initiatives and municipal policymakers.

These idealizing narrative interactions about the ‘strengths’ of Lowenthal and Perry show that Lowenthal's story became meaningful because it connected to the master narratives about welfare transitions, whereas Perry's story remained unintelligible and was not retold. This example shows that idealizing narrative interactions do not promote equal participation and democracy in the development of understandings about participation society and decisions in local enactments of welfare reforms. The stories of the driver and of Perry remained powerless and did not influence decisions about how to allocate care and support, whereas Lowenthal's story was retold on several occasions and had the power to mobilize municipal support for the new services and care point.

### 3.2 Pragmatizing narrative interactions

In pragmatizing narrative interactions, leaders of social care initiatives and municipal policymakers not only celebrated successes of welfare reform and participation but also gave prominence to complex and ill‐fitting stories about vulnerabilities and care. While master narratives guided experiments with new caring environments during transitions, pragmatizing narrative interactions changed understandings about the central actors engaged in giving and receiving care. They challenged relocations of care ‘away from professionals’ and the assumptions about volunteers as embodiments of citizen power. These adjustments in the understandings about participation society also influenced decisions about the allocation of responsibilities and resources for care.

#### 3.2.1 Adjusting narrative relocations of care

Pragmatizing narrative interactions lead to re‐recognition of the value of professional care in the initiatives. In the social care initiatives, it became clear that the often unpredictable needs and vulnerabilities of the growing numbers of participants required significant time and effort. While participants and staff members often managed to support each other in daily work, sometimes personal problems and needs disrupted working relations. The farm (B), for example, had grown within one year from around eight volunteers to around 90. Most of these volunteers struggled with health problems and financial worries. At some point, the farm management started saying that their support was limited. The assistant manager for animals always gave much attention and support to volunteers, but she felt it had reached a limit:

Yes, I want to be supportive, and I want to help, but I don’t know how to help. Because I don’t know in what way I can best do that. And sometimes I would fret over this in the evening after work.

The overburdening of staff and volunteers led to conflicts, intimidation, gossip and dropouts. Two volunteers said, for example, that they felt frightened by the stories told by another volunteer:

We had a girl walking around here… she did everything for the farm manager, morning ‘til evening, and that was sparking off a lot among volunteers, so much gossip and lies, no one dared say anything anymore.

Some conflicts between volunteers led to dropouts who expressed their anger in meetings with the municipality and partner organizations. Even if most volunteers found a valuable and supportive environment in the farm, the ideal of the ‘natural caring environment’ was shattered in these stories. The managing board of the farm invited a municipal official to take part in conversations about these developments. What was needed to improve care? The board did not want to transform the farm into a professional care institute. However, the board and municipal official did conclude that the needs of volunteers required better recognition and care and that the small number of overburdened staff had to be protected. With municipal support, the board of the farm installed a new professional who had studied psychology to improve care at the farm. She was called a ‘volunteer broker’ to avoid an emphasis on psychological problems. This term maintained the connection to the transition narratives that emphasized power and trust in the volunteers but also gave more acknowledgement of their needs. The volunteer broker explained that, instead of doing therapeutic sessions, she focused on the daily ‘lifeworld routines’ of volunteers:

It doesn’t work here to just sit with people and talk for an hour… here, you can just follow people, how they are doing in daily work.

This way, the volunteer broker allowed needs and questions about care and support to surface ‘naturally’ in daily work contacts. Other initiatives also introduced new professionals to deal with the vulnerabilities of participants: a ‘community facilitator’ (C) and an ‘action coordinator’ (A & D). These new professionals were positioned as ‘lifeworld professionals’ within daily personal interactions and not within bureaucratic regimes. Thus, pragmatizing narrative interactions changed the master narrative about welfare reforms: while the master narrative about care within lifeworlds kept guiding new care practices, it now included stories that acknowledged the importance of professional care and support. The adjusted master narrative about participation society also mobilized new financial resources to pay for professional care and support.

#### 3.2.2 Adjusting stories of citizen power

Pragmatizing narrative interactions adjusted master narratives about volunteers as the embodiment of ‘citizen power’. The stories of volunteers in social care initiatives show that problems did not disappear when only emphasizing strengths. Volunteers helped in daily work and care, but they also struggled with personal problems, and this affected other volunteers and staff. Initiative D, for example, developed a restaurant and various production lines in denim and furniture. One of the participants, Mr Pereboom, was an upholsterer who had to quit his job because of deteriorating eyesight and back problems.

I was on sickness benefit. I was… in bed and did not come out anymore. And with this came a lot of stress and heart problems, you name it. Financially, I went back, got into a lot of trouble. On a reintegration trajectory, I came here. I was also pretty depressed, and here, I found a warm welcome. And here I can be myself and work with pieces of furniture, and yes, I also supervise the boys and girls here in doing nice things, and when they have a smile on their face, then I also smile.

While this looks like a story that supports the master narrative about volunteers taking over care and other work from professionals, the coordinator of D showed another side to the position of volunteers in care.

Well, supervision… in technical things he teaches people the principles yes… [but] financially he has had a very difficult time, for a long time, and he borrowed money here to buy fabrics… that became a problem… I had a gut feeling that something was wrong, so I became stricter, and then it escalated… he could not account for his expenses and then the bomb exploded… We left him for a while, and then I called him and said, “yes, this happened and I understand that this is very difficult and that you are financially in a tight place… and when worst comes to worst, I want to hear that, and not in this way… that is not fair to us”.

These interconnected stories show that volunteers, in sharing their ‘strengths’, also share their personal struggles. To articulate the personal struggles of volunteers in the public stories about initiative D, the leaders explained in meetings with partner organizations and the municipality that the meaning of ‘volunteer’ had changed: the initial volunteers just came to help and had no special needs, but the new volunteers came with personal struggles while also wanting to contribute. The reforms created new volunteers, for instance, volunteers who had previously been labelled with low IQ and surrounded with professional care. Many of these new volunteers had lost their way in the changing welfare landscape and needed intensive support. The leaders considered the word ‘participants’ more fitting than the word ‘volunteer’ to recognize that all those joining the initiative were struggling with something. This way social care initiatives adjusted understandings of policymakers and other influential partners about helpful ‘volunteers’: volunteers formed a valuable resource in new care practices, but they were also in a precarious position themselves and in need of care and support. This change in understandings cannot be linked to concrete policy decisions. However, the adjusted narrative about volunteers and ‘citizen power’ did affect decision making and negotiations about the allocation of resources for care and support.

## 4 DISCUSSION

In Western welfare states, people who struggle with disease, disability and unemployment have seen dramatic changes in the forms of care and support available to them, and many discussions deal with the democratic character of these reforms. According to reform advocates, care is moving out of bureaucratic systems and into the lifeworld, and in the process, citizens are liberating themselves from colonizing professional regimes and labels.^1^, ^10^, ^11^, ^12^, ^13^ Advocates also argue that reforms are part of a bottom‐up, citizen‐initiated movement, and they have defined citizen‐initiated change as part of do‐democracy in which citizens gain influence by doing and taking responsibility for the organization of public resources for care and support.^15^ According to critics, however, these reforms have marginalized valuable practices of professional care as well as the people on the receiving end of care.^40^, ^41^, ^42^ They point out that many so‐called citizen initiatives were initiated top‐down by government actors or included only the happy few of well‐off citizens.^3^, ^4^, ^19^ In their analysis, the so‐called do‐democracy is rather a disguise for top‐down management of responsibilities in care. In addition to do‐democracy, welfare reforms were accompanied by experiments with ‘deliberative democracy’, in which random or focused selections of citizens are invited to deliberate about public issues.^5^, ^8^, ^21^ Scholars like Fraser and Polletta, however, point out that the specific types of discourse in deliberative democracy may also exclude the experiences and perspectives of people in marginal positions who have other ways of expressing themselves and have difficulties convincing other ‘deliberators’.^22^, ^23^ As both do‐democracy and deliberative democracy provide highly educated social groups with more power and influence, they are at risk of reproducing or even aggravating social inequalities. Our analysis adds a new perspective to this debate, showing the narrative dimensions of democracy. Many of the actors involved in newly developing care practices during welfare reforms do not participate in roundtable discussions and extensive deliberations. However, they do tell small stories in everyday care settings, in which they organize experiences and make connections with other stories. These small stories may be fragmented and incomplete. However, if they are picked up and retold by leaders of initiatives and policymakers, they gain additional meaning and influence. This focus on narrative interactions enables insight into what happens with the experiences and perspectives of central actors in newly developing care practices, including people in precarious and marginal positions.

Our analysis shows that democratic participation was constructed or obstructed through narrative interactions, depending on the ways in which stories travelled or failed to travel through different narrative environments. Narrative interactions stimulated new public understandings about social care and influenced decisions by including and excluding diverse stories. In idealizing narrative interactions, only the small stories that supported master narratives about welfare reforms were collected and retold by leaders and policymakers. Other ‘mis‐fitting’ small stories were not retold in the narrative environments of leadership meetings and policy meetings. These idealizing narrative interactions can be seen as part of the activist strategies of the leaders of social care initiatives to promote change and find financial and ideological support. In addition, for municipal policymakers, idealizing narrative interactions were strategic as they presented proof that participation society works, and that ‘old’ bureaucratic care systems need to change. However, idealizing narrative interactions also obstructed democratic participation in the development of understandings about welfare reforms, as many stories did not reach a broader public or audience of policymakers. In idealizing narrative interactions, leaders and municipal policymakers did not collect stories about inadequate care in a ‘lifeworld setting’, such as that of the driver who felt uncomfortable with his caring role. Furthermore, they did not retell the stories about the failures of participants to find employment in spite of great efforts and belief in their own powers. These stories remained powerless or even incomprehensible in a setting that celebrated lifeworlds and citizen power.

In pragmatizing narrative interactions, leaders of social care initiatives and municipal policymakers also retold the disconcerting small stories about experiences that did not fit the ideal of participation society. While still connecting to the master narrative, these stories also changed its meaning. ‘Lifeworld care’ and ‘citizen power’ remained central themes, but the conflicts that occur in lifeworlds and the struggles and vulnerabilities of people were taken more seriously. For example, the small stories of angry volunteers and conflicts at initiative B were extensively retold and examined by leaders of the initiative, who also invited a municipal policymaker to this process. This resulted in renewed acknowledgement of the need for professional care. While initial master narratives about participation society contrasted lifeworld care with systems of professional care, these pragmatizing narrative interactions produced a new type of ‘lifeworld professional’ and resulted in concrete decisions about the allocation of financial resources for care. Other pragmatizing narrative interactions about vulnerabilities and personal struggles informed political understandings about the growing group of volunteers engaged in social care initiatives. These volunteers not only constituted proof of citizen engagement but also showed that during welfare reforms many vulnerable citizens were looking for social care environments that could support them in their personal struggles. In this way, small stories that did not fit the master narrative of the participation society gained power through pragmatizing narrative interactions: they affected a change in public understandings of needs, vulnerabilities and adequate care, and they affected decisions about the allocation of financial resources for ‘lifeworld care’, including lifeworld professionals.

The initial master narrative of welfare reforms supports understandings about care in oppositions between professional systems and lifeworlds or dependency and citizen power. Here, increased independence is seen as a sign of citizen power. By contrast, the adjusted narratives produced new discursive spaces that are more inclusive to people in vulnerable and marginal positions. In pragmatizing narrative interactions, participants, leaders, policymakers and partners noted that needs and vulnerabilities do not disappear when medicalizing labels are removed; they are just expressed in new ways. The adjusted master narrative did not promote ideals of independency, and instead formulated ideals of active participation in webs of care and dependency: care and dependency are not the exception but the rule, even if people need different forms of care and some need more than others. This way, the adjusted master narrative about welfare reforms pushed back against increasing marginalization and promoted more equal recognition.

This analysis also adds a new perspective to the literature about the democratization of care. Where most scholarship in this domain has focused on the design of formal procedures and governance structures including clients and lay persons, our focus has been on the influence and translation of stories shared in daily interactions.^43^, ^44^, ^45^, ^46^ Making sense of chaotic, disturbing or isolated stories in the narrative environment of work floors, leadership and municipal policy helps to construct democratic routes in care, as introducing such stories in formal and informal settings implies that the experiences of participants not included in roundtable discussions will be voiced.

## CONFLICT OF INTEREST

The authors have no conflict of interests to declare.

## Data Availability

The data that support the findings of this study are not publicly available due to restrictions, for example their containing information that could compromise the privacy of research participants.
